# Cheating hypnos: can polyphasic sleep schedules reduce the need for sleep?

**DOI:** 10.1093/sleep/zsag058

**Published:** 2026-02-27

**Authors:** Yevgenia Rosenblum, Martin Dresler

**Affiliations:** Donders Institute for Brain, Cognition and Behavior, Radboud University Medical Center, Nijmegen, The Netherlands; Donders Institute for Brain, Cognition and Behavior, Radboud University Medical Center, Nijmegen, The Netherlands

Sleep is a weird behavior: we spend a large fraction of our time in a state of complete neglect of a potentially dangerous environment. The fact that sleep is nevertheless a ubiquitous phenomenon in the animal kingdom suggests that it fulfills functions of vital importance for health and survival, thereby overcompensating for its obvious disadvantages. The multiple functions of sleep are becoming increasingly clear over recent years: Sleep helps to regulate energy and plays a role in endocrine and immune regulation; it supports biosynthesis and the repair of damaged tissue, and clears toxic metabolites that build up during the day. Beyond such basic physiological housekeeping, sleep helps to regulate the experience of stress and emotions, supports memory formation and neural plasticity, and restores attention and executive functions. Even the subjective side of sleep—dreaming—may have biological value, offering a simulation of reality that lets us practice evolutionarily important skills for dealing with threats or social situations [1].

**Figure 1:**
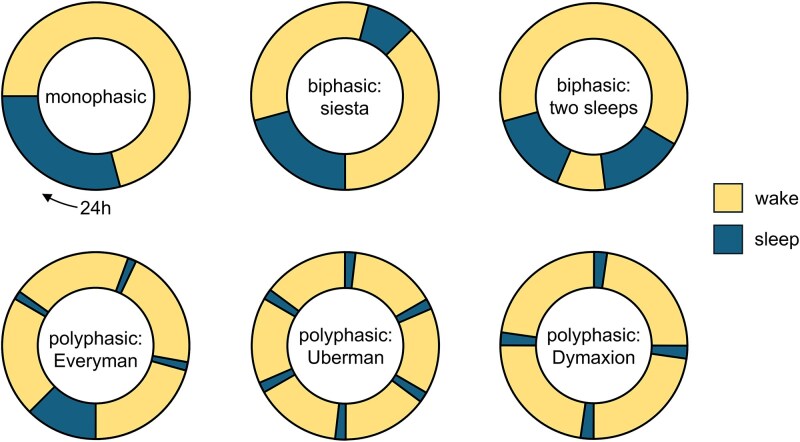
Sleep schedules. Most humans live on a monophasic sleep schedule (top left), or—in siesta cultures (top center)—on a biphasic schedule. There is also historical evidence for biphasic sleep cultures with two separate night time sleep periods (top right [14]). While polyphasic sleep schedules are the natural default in many animal species, in humans, they appear to be a rare necessity to cope with situations of high demands of continuous wakefulness such as ocean races or armed conflicts; however, they can also be a strategic choice to reduce cumulative time spent asleep. The most widely discussed polyphasic schedules for radical sleep time reduction are the “*Everyman*” schedule comprising three brief daytime naps in addition to one more extended sleep period during the night (bottom left); the “*Uberman*” schedule of six 20-minute naps evenly distributed across the day (bottom center); and the “*Dymaxion”* schedule of four 30-minute naps evenly distributed across the day (bottom right [15]).

In competitive job markets and meritocratic societies, productivity and efficiency are strategic advantages for career success. Many people therefore restrict their sleep to maximize time spent awake and thus seemingly productivity, despite the vital importance of appropriate sleep duration, quality and regularity for health and wellbeing. Anecdotal evidence in popular culture suggests that splitting sleep into multiple short segments may enhance daytime productivity: such *polyphasic sleep schedules* are proposed to dissipate homeostatic sleep pressure experienced under sleep deprivation more efficiently than longer bouts of continuous sleep (Fig. 1).

Overall, scientific evidence on the effects of polyphasic sleep restriction is scarce. While many studies have shown positive effects of daytime naps, e.g. on cognitive performance, in particular under conditions of restricted night-time sleep [2], only few studies have tested the effects of more radical forms of polyphasic sleep. Observational research on sailors in ocean races has suggested that under extended high-demand conditions, performance suffers least if restricted sleep opportunities are split into several naps instead of one continuous sleep period [3]. Radical polyphasic sleep schedules generalize this idea: the *Uberman* variant proposes a schedule where sleep is split into six 20 minute naps spaced evenly across 24 hours, resulting in a cumulative sleep amount of only 2 hours per day (see Fig. 1). Empirical data on the effects of such sleep reduction strategies are so far restricted to case studies, where single volunteers have been monitored for some days or weeks on radically polyphasic schedules such as the *Uberman* [4–6].

Summarizing the state of research on polyphasic sleep, a report of the US National Sleep Foundation found no evidence supporting benefits from following polyphasic sleep, and thus disencouraged adopting such schedules due to significant concerns about mental and physical health risks and impaired daytime functioning [7]. Sufficiently powered studies testing the more extreme forms of polyphasic sleep schedules, however, are still lacking.

In this issue of *SLEEP*, Koa & Lo [8] report a study including 40 healthy participants who were restricted to a 2 hour sleep period over 24 hours, either monophasically in the form of a continuous 2 hour nap, or polyphasically via the *Uberman* sleep schedule. By combining polysomnography with a battery of cognitive and psychological tests, the authors compare the two short sleep groups with each other as well as with another monophasic group of 20 participants who had a monophasic 8 hour sleep opportunity.

Compared to the normal sleep group, both short sleep groups experienced greater subjective sleepiness, poorer vigilance and lower positive mood. In the morning, when polyphasic sleepers had cumulated only half the sleep of the monophasic short sleep group, their vigilance impairments were slightly more severe. These findings replicate broadly documented reports on detrimental effects of short- and long-term partial and total sleep deprivation on cognitive performance, mood, and vigilance, both subjectively and objectively [6, 7, 9, 10].

Of note, Koa & Lo found that polyphasic sleepers had lower sleep efficiency and higher proportions of light vs. deep sleep compared to monophasic short sleep. This contradicts the idea that polyphasic sleep may be a hack to make sleep more efficient and thus to compensate for the quantitative loss in total sleep time. Arguably, multiplying sleep opportunities by polyphasic sleep schedules also multiplies the “transition costs” of falling asleep: instead of a single period of winding down for sleep (and complementary: periods of sleep-inertia after awakening!), polyphasic sleepers have to undergo transitions from full wakefulness to sound sleep and back repeatedly, thereby reducing the opportunity for consolidated and in particular deep sleep compared to monophasic sleep schedules. Accordingly, time spent asleep relative to time spent in bed is reduced, while pre-/post-sleep periods for winding down and overcoming sleep inertia accumulate.

The reduced amount of sleep inherent to radically polyphasic schedules such as the *Uberman* raises serious concerns regarding the different functions of sleep. For example, immune regulation or brain clearance crucially rely on slow-wave sleep, so its persistent reduction risks to accumulate external pathogens or internal toxic metabolites such as amyloid-beta, thus increasing the likelihood for developing infectious or neurodegenerative diseases [11, 12]. The *prima facie* evolutionary downsides of sleep as a state of extended neglect of the environment suggest that any option to reduce the time we need to spend in sleep to fulfill its functions would bring considerable adaptive benefits, and would thus likely have been evolutionarily selected for.

This does not implicate that *all* functions of sleep require the same amount of sleep though—different sleep stages and microprocesses may underlie different functions of sleep, each potentially with their own quantitative requirements. Accordingly, some functions of sleep may suffer less from radically polyphasic sleep schedules than others. Our own research on polyphasic sleep illustrates this possibility [6]: a polyphasic sleeper who spent 5 weeks on the *Uberman* schedule showed surprisingly few cognitive impairments; however, he almost completely stopped releasing growth hormone, suggesting that endocrine functions were considerably more severely impaired than cognitive functions of sleep.

It should be stressed that the participants of Koa & Lo experienced polyphasic sleep for a period of only 24 hours. Both internet lore as well as our own research [6] suggest that polyphasic sleepers can adapt to at least some of the detrimental effects of the severe sleep restrictions associated with the *Uberman* schedule. It is thus possible that sleep latency and sleep efficiency would improve over a longer study period. However, investigating radically polyphasic sleep over extended periods with sufficient sample sizes is not trivial: In our previous study, 9/10 volunteers quit the polyphasic schedule during the first three weeks due to its strong impact on their social life and subjective wellbeing, despite their originally strong intrinsic motivation [6]. It seems thus likely that a relatively uncommon level of resilience is required to sustain radically polyphasic sleep schedules for longer periods, making them – independent of general health risks – unsuitable to the broader population.

While research into individuals capable of sustaining polyphasic sleep restriction for longer periods faces obvious issues regarding generalizability, it may nevertheless help to map the richness of sleep physiology to the different functions of sleep, both quantitatively and qualitatively. Given the burden and expected drop-out rates when adopting radically polyphasic sleep, a more promising way to further investigate such schedules may be citizen science approaches [13]: many online communities self-experiment with different sleep hacking strategies anyway, and recent developments in wearable technology allow self-tracking of sleep and other physiological processes with increasingly decent data quality. Academic sleep researchers may learn from such lived experience, and in turn provide citizen neuroscientists with best practices for safe and reliable self-measurement. While it seems unlikely that any sleep hacking trick will allow reducing sleep need to a considerable degree without negative side effects, we may gain new insights into the mechanisms of how sleep benefits our health rather than being “just a bad habit” [14] that wastes our time.
